# Nutritional Intervention and Musculoskeletal Health in Chronic Kidney Disease

**DOI:** 10.3390/nu17050896

**Published:** 2025-03-04

**Authors:** Diana Moldovan, Crina Claudia Rusu, Alina Ramona Potra, Dacian Tirinescu, Maria Ticala, Yuriy Maslyennikov, Andrada Alina Bărar, Alexandra Urs, Ina Maria Kacso

**Affiliations:** 1 Department of Nephrology, ‘‘Iuliu Hatieganu” University of Medicine and Pharmacy Cluj-Napoca, 400012 Cluj-Napoca, Romaniaalina.potra@umfcluj.ro (A.R.P.); tirinescu.dacian@umfcluj.ro (D.T.); cosa.maria@umfcluj.ro (M.T.); maslyennikov_yuriy@elearn.umfcluj.ro (Y.M.); barar_andrada_alina@elearn.umfcluj.ro (A.A.B.);; 2 Nephrology Clinic, Emergency County Hospital Cluj-Napoca, 400012 Cluj-Napoca, Romania

**Keywords:** chronic kidney disease, mineral and bone disorders, osteoporosis, sarcopenia, lifestyle, nutritional intervention, antioxidants

## Abstract

Chronic kidney disease (CKD) is a leading condition in terms of prevalence and overall health impact. With the increased life expectancy of the CKD population and the improvement in medical care, controlling musculoskeletal complications remains a tough challenge. Patients with CKD are prone to falls, fractures and sarcopenia, enhancing the risk of death. A multitude of mechanisms contribute to fractures, and treatment is suboptimal; therefore, prevention must stand out as a key step. This review aims to provide an overview of the most relevant data regarding the impact of nutrition on bone disorders and sarcopenia in CKD. The newest relevant studies emphasize that plant protein intake is associated with a lower production of uremic toxins, lower serum phosphorus levels, and stronger bones. We conclude that patients with CKD should adopt specific diets tailored to the presence of osteoporosis, renal osteodystrophy, and muscle wasting. Low-protein diets or plant-dominant diets containing an adequate amount of protein could be better choices for predialysis patients with CKD in order to protect their bones and muscles, whereas in the dialysis population, a higher protein intake could be essential to prevent osteoporosis and sarcopenia. In all patients with CKD, focusing on antioxidant food intake could provide a strong antiaging benefit through ensuring good musculoskeletal health.

## 1. Introduction

### 1.1. CKD—Epidemiology

We are living in a time in the history of medicine when non-communicable diseases have the greatest impact on survival, and among these, chronic kidney disease (CKD) is one of the most prevalent [1]. It is currently estimated that over 850 million persons have CKD worldwide [2], and as a result of the population aging, prediction data indicate that CKD will become the fifth global cause of death by 2040 and the second cause of death in countries with a long life expectancy by 2100 [3]. Moreover, the number of patients with end-stage renal disease (ESRD) is rapidly increasing, and these patients are confronting the most severe outcomes. Kidney transplantation, the best option for renal replacement therapy (RRT), is hampered by a shortage of kidney donors; therefore, a high demand for both hemodialysis (HD) and peritoneal dialysis has been registered in ESRD [3]. It is unfortunate that the survival benefit in chronic dialysis compared to conservative management is overshadowed by the multitude of uncontrolled chronic symptoms and complications.

### 1.2. Bone and Muscle Health

The increasing life span of the global population has resulted in high interest in physical fitness and the prevention of disabilities. Yet, older adults struggle with frailty rather than enjoying mobility and health. In patients with CKD, musculoskeletal complications are common across all stages; however, the worst situation is observed in patients with ESRD when bone and muscle health suffering become difficult to tolerate [4]. Advancements in treatments for osteoporosis and other bone disorders come as a response to the need for autonomy and for the overall well-being of every person, even in elderly individuals [5]. However, in patients with CKD, none of the available treatments can halt bone suffering, and it is even more problematic that musculoskeletal disorders are associated with prolonged hospitalization and high mortality. One of the main risk factors for these negative outcomes is malnutrition [6].

### 1.3. Nutrition and CKD

The development of treatments for osteoporosis and sarcopenia is remarkable, but at the same time, we are witnessing increasing awareness regarding the role of lifestyle interventions as preventive measures. The progress has been slow and focused on removing unnecessary restrictions, mainly for food with clear health benefits for the general population. Natural bioactive compounds could be useful in treating CKD and can improve body composition in favor of muscles and healthy bone structure [7]. Studies on the impact of variable nutritional patterns on the musculoskeletal system in patients with CKD have provided conflicting results. Some of them revealed a relationship between different nutrients with better bone mineral density (BMD) or bone biopsy features, while others found a worse significant association [8,9]. The aims of this paper are to review the current knowledge about the interactions of nutritional intervention with bone disorders and sarcopenia in patients with CKD and to conclude what the best food choices are to achieve musculoskeletal strength.

## 2. Musculoskeletal Involvement in CKD—Pathogeny

### 2.1. Chronic Kidney Disease—Mineral and Bone Disorders (CKD-MBD)

The term CKD-MBD refers to a systemic syndrome involving mineral abnormalities of calcium (Ca), phosphorus (P), parathyroid hormone (PTH), or vitamin D levels, leading to bone complications vascular calcification due to CKD [10,11]. Renal osteodystrophy is a complex of different bone diseases in CKD, defined according to imagistic and histological abnormalities. There are several types of morphological bone modifications in CKD-MBD depending on turnover, mineralization, and strength. The remodeling process consists of the formation and resorption of bone tissues depending on the balance of osteoblast and osteoclast activities [8]. Renal osteodystrophy includes four entities, namely fibrocystic osteomalacia, adynamic bone disease, and mixed lesions. Fibrocystic osteitis is associated with high bone turnover secondary to hyperparathyroidism, while osteomalacia is the consequence of vitamin D deficiency, and adynamic bone disease occurs due to low bone turnover, which is frequently seen in patients with CKD with low PTH, particularly after parathyroidectomy [12]. The pathogeny of CKD-MBD implies the dysregulation of Ca and P, which depends on the action of parathormone (PTH), of the nutritional and active forms of vitamin D, and of the FGF23-Klotho axis and their impacts on bone. The body systems involved in this regulation process are the parathyroid glands, the small intestines, the bones, and the kidneys. They all interact to maintain normal serum levels of Ca and P [13]. The clinical impact of this interplay is important in CKD, especially in advanced stages. In chronic HD, increased mortality was reported in patients with high P and Ca levels accompanied by high or low PTH levels [14].

In CKD-MBD, different factors contribute to frailty, such as uremic toxicity [15] or metabolic acidosis [16]. The amount of acid produced in the organism needs to be buffered, but in CKD, there is a decreased ability to generate bicarbonate. Studies have shown that acid loading adversely affects phosphorus homeostasis [16]. Prolonged metabolic acidosis decreases bone strength and favors fracture risk due to increased osteoclastic activity, and due to malnutrition [16].

Other important contributors to CKD-MBD are inflammation, oxidative stress, and mitochondrial dysfunction. CKD-MBD and malnutrition–inflammation complex syndromes are often associated in chronic dialysis patients, leading to a vicious cycle in which each perpetuates the other’s progression [17].

### 2.2. Osteoporosis

Osteoporosis is one of the most common systemic bone diseases in clinical practice and mostly occurs in postmenopausal women [18]. Osteoporosis is a degradation of bone tissue structure with a decrease in the BMD, bone fragility, and high risk of fracture. About 9 million osteoporosis-related fractures are globally reported every year [5,18]. The pathogeny of bone loss and fracture in CKD is complex and multi-factorial, as bone strength is compromised due to the deterioration of both bone quantity and quality [19]. This could be an explanation for the difficulty of reaching a consensus on the optimal diagnostic method of osteoporosis in patients with CKD. As a result, osteoporosis assessment in CKD may include dual-energy x-ray absorptiometry (DXA) or quantitative computed tomography for quantity and imagistic trabecular bone score or bone biopsy for quality [20].

### 2.3. Sarcopenia

Sarcopenia is a progressive muscle disease characterized by loss of muscle mass, muscle strength and physical performance. Sarcopenia is age-related, has a high prevalence in CKD, up to 19%, and becomes more prominent as CKD progresses [21]. In children with CKD, the muscle–bone axis influences growth, and it is responsible for their muscle mass loss. Mechanisms such as systemic inflammation, anorexia, physical inactivity, vitamin D deficiency, impaired growth hormone/insulin growth factor 1 axis play important roles in physical underdevelopment of ESRD children [22]. The pathophysiology also involves ubiquitin, myostatin, uremic toxicity, reduced energy or nutrient intake, altered contractile activity, reduced myogenesis and reduced protein synthesis. All of these contribute to muscle degradation and impaired regeneration [23]. The theory of the functional muscle–bone unit has a physiological basis, and it is supported by two recent studies showing positive correlations between skeletal and, respectively, psoas muscle mass index with BMD in HD patients [24,25].

**Figure 1:**
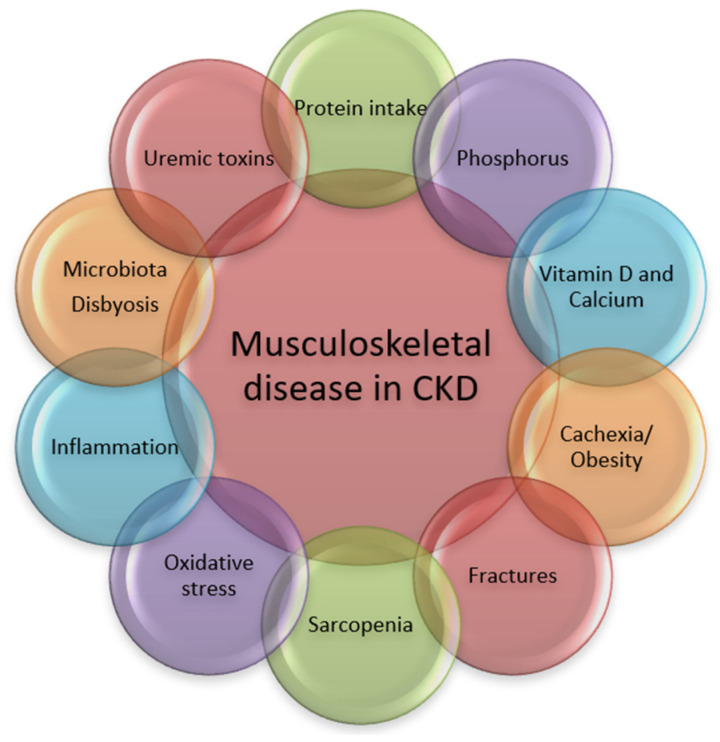
Pathogenic pathways associated with musculoskeletal disorders in CKD.

The pathogenic pathways leading to musculoskeletal disorders in CKD are numerous and the process is complex and multifactorial (Figure 1).

Intact bones, joints and muscles, good physical performance and the absence of specific symptoms, signs, laboratory and imagistic modifications are central to musculoskeletal health.

## 3. Impact of Musculoskeletal Disorders on Outcome in CKD Patients

### 3.1. Clinical Impact of CKD-MBD

As CKD progresses, more patients develop bone and mineral metabolism disorders. Clinical manifestations of CKD-MBD consist of bone pain, muscle–tendon ruptures, pruritus, increased incidence of fractures, bone deformities and growth arrest in children. The reported global incidence of falls in CKD patients is far higher than the general population. Approximately 30–60% of dialysis patients experienced falls, and of these, 16% resulted in fractures and 4% in death [26]. Fractures create a vicious cycle, reducing physical activity, which consequently causes muscle weakness, fear of falling, further decreased physical activity and, eventually, higher risk of falling [12,27]. Good physical function is a prerequisite for being involved in social and other different daily life activities. One of the main goals in the care of CKD patients in all stages is to prevent disability, because many patients with CKD-MBD experience functional impotence and lower quality of life. An accurate and feasible assessment of the CKD patient must include the evaluation of physical function [28]. CKD-MBD is an important risk factor for mortality and CKD progression leads to risk augmentation [29,30]. Mortality in HD patients with fractures is significantly higher than in those without fractures [31]. Therefore, measures to prevent fractures need to be actively implemented.

### 3.2. Clinical Impact of Osteoporosis in CKD

A Canadian study on 19,973 individuals analyzed the relationship between frailty and bone health. Frailty consists of at least three of these five symptoms: weakness, slow walking speed, low physical activity, exhaustion, and unintentional weight loss. The study demonstrated that having at least one frailty criterion was associated with a higher risk of fracture and a lower BMD [32]. CKD may be considered a risk factor for fractures, as low BMD in patients with CKD stages 3a–5D has been linked to a 1.5- to 2-fold higher fracture risk compared with BMD-matched patients without CKD [20]. Concomitant loss of BMD, bone strength, muscle mass and strength in CKD may lead to mechanical impairment of the muscle–bone axis with detrimental impact on social roles and, consequently, low quality of life [32].

### 3.3. Clinical Impact of Sarcopenia in CKD

CKD is responsible for muscle mass loss, with a great influence on physical performance. CKD also interferes in the biomechanical communication between muscles and bones, with an impact on outcomes. A recent registry study on a large number of adult Korean HD patients demonstrated that lean body mass has a significant role in predicting mortality in HD patients across different age groups [33].

## 4. Management of Musculoskeletal Disorders in CKD

There is a global focus on the goal of extending dialysis-free time and delaying dialysis initiation in CKD populations, with a secondary benefit in preventing some of the musculoskeletal problems [34]. However, specific approaches are needed. Kidney Disease Improving Global Outcomes (KDIGO) 2017 Clinical Practice Guideline for CKD-MBD has recommended thorough treatment decisions for the various typical bone diseases in CKD patients, but also for osteoporosis [10,11,14].

### 4.1. CKD-MBD Treatment

The current approach for CKD-MBD targets mainly hyperphosphatemia, hypocalcemia and vitamin D deficiency. The control of secondary hyperparathyroidism is the cornerstone for the management of CKD-MBD. Evidence has indicated that hyperphosphatemia is “a toxic factor” at multiple levels, and it has been associated with severe complications and increased mortality [12]. In controlling hyperphosphatemia, phosphorus binders plays a key role in eliminating the gastro-intestinal load. The range of agents extends from calcium-based (Ca carbonate or acetate) to noncalcic phosphate binders (sevelamer, lanthanum, or sucroferric oxyhydroxide). The first category can increase the risk of vascular calcification, and the second can be used only in advanced CKD stages. As for vitamin D use, in all stages of CKD, serum calcidiol should be maintained within the normal range. The use of calcitriol or any other active analogs is not routinely recommended, being reserved for severe hyperparathyroidism cases. For ESRD patients in chronic dialysis programs, calcimimetics are useful treatments for secondary hyperparathyroidism [13]. Adequate dialysis is essential in controlling CKD-MBD in CKD stage 5D, with the potential to eliminate phosphorus and normalize serum Ca levels. In patients with unresponsive or tertiary hyperparathyroidism, surgical removal of the parathyroid glands should be considered. The current recommendation is to perform parathyroidectomy if PTH levels remain above 800 pg/mL despite adequate medical treatment and if it is accompanied by clearly related signs and symptoms [11].

### 4.2. Osteoporosis Treatment

Some currently prescribed drugs for osteoporosis may not be suitable for CKD patients. The first-line anti-osteoporosis drugs, bisphosphonates, may worsen kidney function or, in ESRD, are relatively contraindicated. Denosumab, a humanized monoclonal RANKL antibody, is an antiresorptive factor not cleared by the kidney and has been used for treatment of osteoporosis, with positive effects in terms of BMD and fracture incidence. An interesting study has proven the beneficial effect of denosumab for muscle and bone tissue in osteoporotic mice and female humans [35]. It can be safely used, but in ESRD, there are concerns about the risk of severe hypocalcemia [20]. Romosozumab, a humanized monoclonal antibody and inhibitor of sclerostin, has been proven to cause a significant change in BMD in all kidney function categories [36]. Teriparatide, an anabolic, bone formation drug, is not recommended is most cases, being of special use in parathyroidectomized patients or those with high suspicion of low bone turnover disease. A recent meta-analysis highlighted the effectiveness of anti-osteoporotic agents (teriparatide, denosumab, romosozumab, raloxifene) in lowering vertebral fracture risk in CKD patients, particularly in stages 1–3, with no benefit in stages 4 and 5 [36]. The absence of reported side effects encourages further research, especially because advanced CKD patients encounter the lowest BMD, and the needs are not yet met.

### 4.3. The Key Therapeutic Options for Sarcopenia

The key therapeutic options for sarcopenia management include one or a combination of exercise, nutrition, and pharmacological interventions. A growing body of literature has reported specific benefits of these interventions in CKD-associated sarcopenia across the disease spectrum [37]. There is a growing interest in the question of combining nutrition and exercise in CKD, and dialysis can improve the musculoskeletal outcomes [38,39].

## 5. Impact of Nutrition on Bone Disorders and Sarcopenia in CKD Patients

Defining healthy nutrition is somehow difficult, because it is a continually changing concept. This happens as a consequence of increasing focus on research in the food domain and due to ongoing study of the effects that different nutrients exert on the human body. Nutrition patterns and individual needs should be adjusted every time new and significant evidence points to a positive influence that a specific food group has on general health and on the prevention of non-communicable diseases, including CKD [40]. Conversely, given the potential of some foods to become risk factors for diseases, the “World Health Organization (WHO) Global Action Plan for the Prevention and Control of Noncommunicable Diseases” included strategies against behavioral risk factors, with a focus on unhealthy diets [41]. For the general population, WHO recommended balancing energy intake, increasing consumption of fruits and vegetables, and limiting the intake of saturated fats, sugar and salt [41].

Nutritional habits contribute to 20–40% of the risk of osteoporosis [42]. Chronic kidney disease is a condition associated with higher rates of osteoporosis than the general population. Therefore, dietary intervention to prevent the osseous manifestations of CKD is even more important. At the same time, optimal nutrition is more difficult to accomplish due to numerous risk factors and restrictions. As for CKD-MBD, the current diet recommendation includes low phosphate intake, reduced dietary acid loads, measures to correct Ca and vitamin D levels [11,43]. The latest guidelines for the management of CKD recommend increased consumption of plant-based foods and decreased consumption of animal-based foods and ultraprocessed foods. Education about dietary adaptations regarding P, Ca, sodium, potassium, protein, and even water intake should be an important step in CKD management. All these measures aim to delay progression to ESRD and to prevent chronic complications, particularly CKD-MBD and osteosarcopenia [34]. Lifestyle interventions such as modifying nutritional habits proved to be effective in reducing risk of vascular calcification [44] and frailty in CKD-MBD [45,46]. The efficacy and safety of these interventions in patients on musculoskeletal involvement in people with CKD are still undetermined. Thus, there is an urgent need for relevant and reliable information on whether specific foods are beneficial for all various bone diseases in CKD patients.

### 5.1. Proteins

A low-protein diet is often recommended to predialysis CKD patients. According to the KDOQI 2020 clinical practice guidelines for nutrition in CKD, daily protein intake recommendation should vary according to CKD stage. In stages 3–5, CKD patients are prescribed low protein intake (0.55 to 0.60 g/kg/day), aiming to postpone dialysis initiation by reducing uremic complications associated with protein catabolism [47]. The alternative is very low protein intake (0.28–0.43 g/kg/day) with keto-amino acid analogues. With respect to the amount of protein intake, the KDIGO guidelines for CKD management updated to 2024 are less demanding, allowing in CKD stages 3–5 a protein intake of up to 0.8 g/kg/day, including at least 50% of proteins of high biological value [34].

Metabolic acidosis is one of the concerns regarding animal protein intake in CKD, due to its detrimental effects on bones and muscles. Food sources of acid are meat, cheese, and eggs, as all these increase the intake and metabolization of acid in the body. On the other hand, fruit and vegetables are good buffers, correcting metabolic acidosis. Contemporary fast-food diets deliver a high acid load, which has to be neutralized by the bones and muscles. Some studies reported that reducing acidic food by replacing meat with vegetal proteins can protect bones and muscles and slow CKD progression [48]. Several benefits emerge from metabolic acidosis correction in adult CKD patients, such as reduced protein degradation and muscle wasting, better serum albumin levels and nutritional status [16,19]. In a clinical setting, the nutritional status of CKD patients is best assessed using questionnaires, biochemical parameters such as serum albumin, cholesterol, even phosphorus and creatinine in HD patients and anthropometric measures and muscle strength. The follow-up is an important step for assessing adherence [34]. In a small study on subjects with CKD stages 3–4, an omnivorous diet containing 70% protein from plants for 4 weeks was efficacious in lowering urine phosphorus and titratable acid. The effect of such a diet was successful on mineral metabolism and acidosis in CKD. Therefore, it should implicitly be helpful in musculoskeletal health, as phosphorus and acidosis are the main pathogenic agents in CKD-MBD [49] (Table 1).

High-protein diets are essential for healthy bones, being associated with higher BMD. Undoubtedly, building or preserving muscle mass starts from a foundation of proteins. Chronic dialysis treatment leads to loss of proteins and increased muscle catabolism; hence, the KDOQI guidelines for nutrition in CKD recommend that patients treated with dialysis be prescribed high protein intake (1.0–1.2 g/kg/day) [47]. Based on the National Health and Nutrition Examination Survey (NHANES) data, Lee et al. evaluated bone densities of different femoral areas according to different protein diets. In subjects without CKD, higher-protein diets led to higher femoral BMD. High-protein CKD patients did not develop higher femoral BMD and neither did those with low-protein diets reduce their femoral BMD. However, CKD resulted as a risk factor for reduced BMD over the intertrochanteric bone region [9] (Table 1). In patients with ESRD, protein malnutrition is common and is usually caused by low-protein diets, CKD progression, and prolonged dialysis treatment. Hemodialysis is responsible for a loss of 3–8 g of aminoacids per session [50]. In a post hoc analysis of the IHOPE trial, HD patients were randomized for 12 months to placebo, 30 g of whey protein supplementation, or protein plus intradialytic bicycling. The study demonstrated that protein supplementation with or without exercise training is associated with improved hip BMD, preventing the loss of bone mass in elderly individuals [51] (Table 1).

Considering sarcopenia, it is common in ESRD, as these patients are highly sedentary and at risk of malnutrition. A recent Australian single-center cohort of maintenance HD patients reported a sarcopenia prevalence of 18%. In this study, low serum albumin and phosphate resulted as significant risk factors for muscle wasting. These findings confirm that protein malnutrition is an important risk factor for sarcopenia [52] (Table 1).

Specific protein consumption has gained impressive popularity in recent years as a complementary approach to body building. There is an ongoing debate about these protein supplements, especially creatine, one of the most commonly used, in regard to beneficial or harmful effects in CKD patients. Creatine is an essential contributor to cellular energy homeostasis. Recent findings suggest that endogenous creatine production progressively decreases with increasing stages of CKD. Creatine deficiency in CKD was associated with fatigue, muscle wasting, impaired cognition, and high mortality. Creatine coming from meat and dairy increasingly becomes an essential nutrient and there is a debate about the fact that newer recommendations for plant-based diets may worsen this deficiency [53]. As might be expected, numerous concerns are addressed to the potential of creatine to impair kidney function. However, a clinical trial consisting of 12-week creatine supplementation showed a significant increase in serum creatinine, while cystatin C remained unchanged [54]. Other studies found no change in serum creatinine after creatine supplementation [55]. Consequently, in CKD patients receiving creatine supplementation, creatinine levels become unreliable in evaluating the renal function and cystatin C clearance should be used to assess glomerular filtration rate [53]. A recent small double-blinded study conducted on 40 HD patients explored the effect of creatine supplementation on body composition and malnutrition–inflammation score. At 1 year, creatine supplementation was associated with increased fat-free mass and skeletal muscle mass, but the authors assume that the simultaneous increase in intracellular water may influence results. There was no attenuation of malnutrition–inflammation score [56]. The results indicated that increasing creatine intake (even without resistance training) enhances fat-free mass and skeletal muscle mass in HD patients, indicating a potential role of creatine in the future nutritional management of sarcopenia in CKD (Table 1). In conclusion, healthy proteins from lean poultry, peas, chickpeas, beans, lentils, and fatty fish are possible choices in CKD for the benefits on muscular and osteoarticular functionality. The amount of protein depends on the CKD stage, and creatine may play a role in the future of CKD-MBD nutrition [57].

### 5.2. Phosphorus, Vitamin D, Calcium and Magnesium

Efforts to control hyperphosphatemia in patients with CKD are essential for preventing secondary hyperparathyroidism and renal osteodystrophy [58]. General recommendations for maintaining serum phosphorus within normal limits include monitoring the dietary intake of phosphorus and taking phosphate binders. Dietary strategies to reduce phosphate levels include abstaining from phosphate-rich foods, as well as paying attention to bioavailability [59]. The dietary phosphorus burden in kidney disease contributes to the pathogeny of CKD-MBD. Dietary phosphate restriction is used commonly to improve outcomes, but the fact that phosphate intake usually parallels protein intake makes the situation more complicated, as CKD patients need to receive an adequate amount of dietary protein to avoid malnutrition [58]. The real restriction should be addressed to phosphate additives. Such inorganic phosphate additives are widely used to produce fast foods, restructured meat, soft drinks, spreadable cheeses, sauces, and frozen bread products [60,61,62]. Sherman et al. measured the phosphorus, potassium, and protein content in uncooked meat and poultry products, and they discovered that phosphorus and potassium content were two- and three-fold higher, respectively, due to additive content, and this information is often lacking from food labels [60].

An interesting experiment was conducted by Gutierrez et al., assessing the impact of additive intake on bone. After an additive-enhanced diet, healthy individuals, but also mice had modified bone biomarkers, and the BMD decreased in mice [63] (Table 1). There is a very fine line regarding the balance of nutrients in food. Many of the natural foods known to be rich in phosphorus were traditionally disapproved in CKD-MBD. The capacity to absorb phosphate should also be considered while deciding on appropriate nutrition, as the intestinal absorptive rate of additive phosphate is over 80%, while for phosphate contained in plants, it is about 30% [64]. Lately, foods like beans and nuts have received more attention for use by CKD patients with the knowledge that the phosphorus is less absorbed. Thus, in predialysis patients, beans and nuts may be more acceptable protein sources, because the phytate and fiber content leads to a lower phosphorus absorption rate [65]. A recent study has evaluated adults’ phosphorus knowledge and dietary intake of phosphorus, hypothesizing that there would be a negative relationship, in which high phosphorus knowledge scores would contribute to lower consumption of phosphorus-rich foods. The results showed no association with phosphorus knowledge scores and dietary intake of phosphorus, indicating a gap in understanding and a need for tailored nutrition education among adults on dialysis [66].

Vitamin D deficiency is common in patients with CKD and associated with poor outcomes. Vitamin D is a well-known modulator of musculoskeletal health in CKD through its effects on Ca, P, PTH and, consequently, on bones [67]. Oxidative stress in CKD is also impactful on bones and exacerbated by vitamin D deficiency. Nonclassical vitamin D actions are thought to be related to effects on uremic inflammatory status, and on immune dysfunction. It was therefore considered as reasonable to improve 25-dihydroxyvitamin D supply for extrarenal production of 1,25-dihydroxyvitamin D. In HD patients, cholecalciferol therapy led to a significant decrease in inflammatory markers and cytokines [68]. Current clinical practice guidelines recommend supplementation with nutritional vitamin D, as for the general population. The deficit is usually corrected with oral medication, but natural sources of vitamin D such as fatty fish, hazelnuts and mushrooms should be seriously advised [69].

There may be a concern that excess magnesium (Mg) may impede bone mineralization. Sakaguchi et al. conducted a study on a large nation-wide database of patients undergoing HD in Japan with no history of hip fracture. The follow-up lasted 2 years and they identified 2% new hip fractures. The risk of hip fracture was not elevated in HD patients with mild hypermagnesemia, but lower serum Mg levels were significantly associated with an increased risk of hip fracture. The population-attributable fraction of serum Mg level for new hip fractures was 13.7% which was much higher than that of serum Ca, P, and PTH levels [70] (Table 1). A higher dietary amount of Mg can be obtained from cashews, peanuts, almonds or spinach intake. Interestingly, there is a significant interaction between Mg and phosphate in terms of absorption and bone effects [71].

### 5.3. Plant-Based Diets and Microbiota

#### 5.3.1. Plant-Based Diets and Plant-Dominant Diets (PLADO)

A plant-based diet leads to a 12% lower risk of glomerular filtration rate decline [72]. Even new CKD cases can be prevented by eating plant-based foods. A community-based prospective Korean cohort study demonstrated that a diet rich in vegetables reduces the incidence of CKD [73]. Individual components of such a diet have a beneficial influence on blood pressure, lipid levels, thrombosis and fibrosis risks, oxidative stress, inflammatory responses, and endothelial function in CKD patients. Plant-based diets were also associated with a better quality of life, better management of complications and decreased mortality in adults with CKD [74,75]. Evidence supports the use of plant-based diets for their survival benefit. Important findings come from a registry study which proved the association between a diet with a higher proportion of protein from plant sources and lower mortality in CKD stages 3a–5 [74]. The consumption of fruits and vegetables in patients with stage 3 CKD is seen as an effective method for correcting metabolic acidosis and preserving renal function [76]. The latest KDOQI guidelines recommend an increased dietary intake of fruits and vegetables in adults with CKD 1-4, to decrease net acid production in order to reduce the rate of decline of kidney function and to protect bones from MBD [47]. Acidemia is a major contributor to bone abnormalities in CKD-MBD. Plant foods contain citrate, which is metabolized to bicarbonate and can help reduce metabolic acidosis [77], leading to improvements in bone metabolism [16]. Among other nutrients, citrate is involved in pathophysiology and the management of bone diseases in CKD. Bone tissue is the main intrinsic source of citrate, being produced by osteoblasts. At the same time, citrate influences osteoblasts’ differentiation and functionality. Increasing consumption of oranges, lemons or other types of citrus fruits with high citrate content may be helpful in neutralizing the acid load, and, consequently, in managing CKD-MBD [78]. A new experimental study demonstrated that diosmin, a bioflavonoid contained in citrus, protects against CKD-induced osteopenia in CKD rats, preserving bone mass and strength [79] (Table 1). Thus, we may assume that citrus consumption may have anti-osteoporosis properties in CKD patients.

In a single-center cross-sectional study on autosomal dominant polycystic kidney disease (ADPKD) patients, higher adherence to DASH diet (rich in vegetables, fruits and whole grains) was associated with low risk of reduced handgrip strength, indicating that the DASH dietary pattern may promote the preservation of muscle strength in ADPKD patients [80] (Table 1).

Yet, plant proteins are less anabolic and less digestible than animal proteins. Accordingly, for a similar anabolic effect on muscles and bones, the adequate amount of plant protein should be higher than the animal proteins [81]. Mansouri et al. evaluated the association between pro-vegetarian dietary patterns and the risk of protein-energy wasting and sarcopenia in CKD patients. Their findings indicated that greater adherence to pro-vegetarian diets was negatively associated with the odds of protein-energy wasting, but no association was shown between these diets and the odds of sarcopenia. Therefore, while these results encourage the consumption of plant-based foods, this dietary pattern may not effectively reduce the risk of sarcopenia in CKD patients, particularly if it causes insufficient protein intake [82].

High-protein plant-based food is advised if the potassium content is not too high [11]. Even regarding potassium-related restrictions, we have new surprising data. In a prospective study on HD patients, decreased dietary potassium intake was associated with higher mortality risk. These findings suggest that excessive dietary restriction may be harmful in HD patients [83]. These may indicate the need for a paradigm shift in judging “bad potassium” indiscriminately.

#### 5.3.2. Uremic Toxins and Microbiota

There is a large body of evidence on the accumulation of uremic toxins in CKD patients and its detrimental influence on bone quality and quantity [84]. In most studies, indoxyl sulfate and p-cresyl sulfate, the most important uremic toxins, reduce bone turnover and suppress bone formation. Uremic toxins play a crucial role in the development of low bone turnover disease in CKD through downregulation of PTH receptor expression on osteoblasts and through induction of oxidative stress. Reactive oxygen species inhibit osteoblast functions and stimulate osteoclast proliferation, induce PTH resistance, contributing to the installation and progression of adynamic bone disease in CKD [85]. The results of Barreto et al. indicate a positive association between indoxyl sulfate levels and bone formation rate, osteoblast surface area, osteoid volume, and bone fibrosis volume [86]. Such findings may be explained by the role indoxyl sulfate plays in the pathogeny of bone resistance to PTH and, eventually, of low turnover bone disease [86,87] (Table 1). The impact of a uremic inflammatory environment is not negligible, with CKD inducing decreased muscle protein synthesis and muscle breakdown.

Microbiota plays a specific role in bone metabolism and function. CKD induces dysbiosis, with consequences for various pathological processes. A recent review highlighted important pathogenetic connections between gut microbiota and bone health in CKD patients, demonstrating how the accumulation of indole and p-cresol in CKD disrupts microbiota and impairs bone formation by inducing PTH resistance in bone cells [88]. The decrease in gastrointestinal absorption and the increase in uremic toxin removal may be important in the treatment of uremic osteoporosis [89]. Probiotics are living microorganisms which restore the balance of intestinal microbiota and improve mucosal integrity. Foods rich in probiotics such as yogurt, kefir, pickles, sauerkraut or kimchi, can mitigate uremic toxicity and protect the musculoskeletal function [90]. Recently, a growing body of evidence has emerged on the benefits of plant-dominant low-protein diet (PLADO) in favoring healthy microbiomes. Adopting PLADO prevents constipation and, as a result, reduces hyperkalemia and relieves uremic toxin burden, with a better preventive effect against metabolic complications in CKD compared to animal protein-dominant intake [91]

A long-standing but untested component of the low-phosphorus diet is the promotion of refined grains over whole grains. Due to the beneficial fiber content of whole grains, this recommended restriction should be thoroughly analyzed. A recent paper reviewed scientific evidence for avoiding whole grains in the dialysis population. Although estimated phosphorus intake was higher, the modification of serum phosphorus was insignificant, concluding that there is no strong evidence in favor of restricting whole grain over refined grain [92].

The attempt to restrict phosphorus intake has led to other inappropriate recommendations, namely, to avoid nuts in CKD. However, less than a third of the dietary organic phosphorus from nuts is absorbed, so it is unlikely that they contribute to dietary phosphorus burden in CKD [93,94]. Various kinds of nuts have high fiber content, with benefits for creating a healthy microbiota. In an experimental model of CKD, a Brazil nut-enriched diet was able to modulate enteric glial cells and gut microbiota [95]. A different study proved the possibility of modulating BMD using a Brazil nut-enriched diet in a CKD experimental model [96].

### 5.4. Energy-Dense Foods

#### 5.4.1. Energy-Dense Diets 

Energy-dense diets are detrimental in regards to CKD, as they promote the main causes of CKD, obesity and diabetes mellitus. The least healthy choice consists of saturated fats. However, reverse epidemiology in advanced CKD links mortality with low body weight, so energy-dense diets may be helpful in preventing weight loss, often an indicator of frailty, which is an important risk factor for falls and fractures. In a group of older patients with advanced CKD, Molinari et al. have demonstrated that frailty is associated with malnutrition–inflammation syndrome, underscoring the importance of addressing malnutrition in order to prevent the onset of frailty in this population [97]. However, returning to its nephrotoxic potential, energy-dense food also has negative consequences on CKD-MBD. It induces decreased absorption and activation of vitamin D, hyperphosphatemia, high levels of FGF23, low levels of Klotho, and, eventually, renal osteodystrophy. Furthermore, obesity is a proinflammatory factor, promoting the progression of CKD and musculoskeletal disease. The implementation of caloric restriction with or without aerobic exercise is feasible and can improve body weight, as well as reduce fat mass and proinflammatory and oxidative stress [98]. We should be aware that dietary recommendations on energy intake should be different according to age, gender and physical activity, targeting an ideal body mass index (BMI)/waist-to-height ratio to be achieved through nutrition [34,47]. The latest KDIGO guideline on CKD management recommends a minimum of 150 min of moderate exercise to supplement the effects of nutrition [34]. Still, not all fat is bad.

#### 5.4.2. Omega-3 Fatty Acids

Omega-3 fatty acids are polyunsaturated fatty acids (PUFAs), the active ingredients of oily fish, with important benefits for general health. An analysis of the NHANES cohort population proved a significant inverse relation between dietary omega-3 intake and all-cause mortality in patients with CKD [99]. Omega-3 fatty acids improve bone quality by promoting bone mineralization and preventing bone decay [100]. Alternative sources can be seafood, sesame, pumpkin chia or flax seeds. Recently, an original study demonstrated the causal beneficial effect of PUFAs on BMD and fracture risk [101]. The influence of omega-3 fatty acids on skeletal muscle protein metabolism and mitochondrial bioenergetics was demonstrated through muscle biopsies [102]. Liu et al. analyzed data from 8889 participants from the NHANES cohort and tested the relationship between the presence of osteopenia or osteoporosis based on BMD scores and dietary omega-3 intake. This study displayed a significant correlation between low dietary omega-3 fatty acid intake and high osteoporosis risk, suggesting that omega-3s play a crucial role in bone health. These findings are exciting, but patients with chronic renal failure were excluded from this study [103].

#### 5.4.3. Olive Oil

Olive oil has been found to improve bone quality, by increasing alkaline phosphatase activity and bone mineralization through its content in hydroxytyrosol. This is a phenolic compound that has antioxidant, anti-inflammatory and antiosteoporotic properties. Olives and olive oil favor maturation of osteoblastic cells and prevent the loss of bone mass induced by inflammation and CKD [43,104].

#### 5.4.4. Nuts

Some specific nuts, such as walnuts, pecan and pistachios, have a high content of omega-3 PUFAs. Nut consumption is another source of healthy fats, with valuable effects on CKD and mortality in the United States [105].

### 5.5. Antioxidant and Anti-Inflammatory Foods

Oxidative stress and abnormal osteocyte apoptosis are often related to dysregulation of bone turnover, and so vegetables or fruits with high antioxidant potential may play an important role in the prevention of chronic bone loss. An exciting new subfield of nutrition addresses antioxidant and anti-inflammatory foods. These are also called antiaging, senolytic, functional foods or superfoods. Redox signaling and inflammatory pathways play important roles in CKD-associated cachexia, sarcopenia and osteoporosis pathogeny [106]. Antioxidant depletion has been involved in chronic diseases and abnormal bone remodeling, which are signs of osteoporosis [107]. Exercise capacity in skeletal muscle is positively correlated with mitochondrial function, which is mainly controlled by mitochondrial biosynthesis and degradation. Therefore, in natural antioxidants lie great potential to reduce inflammation, oxidative stress and to prevent CKD-MBD [108].

#### 5.5.1. Diets Rich in Berries

Diets rich in berries, especially blueberries or bilberries, which contain a large number of phytochemicals, also provide health benefits. The most prominent of these phytochemicals, termed anthocyanidins, have potent anti-inflammatory and antioxidant effects. Data suggests that blueberries might inhibit osteoclastogenesis in model mice and so could also be useful for the prevention of bone loss and osteoporosis. Delphinidin, one of the major anthocyanidins, prevents bone loss through the inhibition of excessive osteoclastogenesis in osteoporosis model mice [109]. Blueberry juice protects osteocytes and bone precursor cells against oxidative stress, partly through the activation of SIRT1; reduced SIRT1 expression has been associated with osteoporotic hip fracture [110]. Various berries (blueberry, cranberry, raspberry, and strawberry) could possibly improve the uremic condition by reducing the levels of uremic toxins via modulation of the gut microbiota and, consequently, protect from bone and muscle loss [111].

#### 5.5.2. Resveratrol Is a Polyphenol

Resveratrol is a polyphenol found in grape skins, blueberries, cocoa, and peanuts and has proven efficacy in reducing reactive oxygen and nitrogen species. There is evidence in favor of grape-based food supplementation to prevent the development of CKD [112]. It can contribute to bone restructuring through enhancing osteogenic differentiation and mitochondrial biogenesis from human periosteum-derived mesenchymal stem cells [113]. Murillo-Ortiz et al. conducted a study on 40 HD patients to test the effects of resveratrol and curcumin supplementation on the recovery of bone and muscle mass and protein oxidation, lipid peroxidation and iron overload. Curcumin is a polyphenolic compound and has been reported to have potential benefits for oxidative stress and inflammatory disease; it has the potential to prevent muscle damage and to increase osteoblast survival by downregulating nuclear factor KB. They proved that gain of bone and muscle mass was possible with combined supplementation with curcumin and resveratrol for 12 weeks [114] (Table 1). A recent study evaluated the effect of polyphenols on physical performance and body composition of 40 sarcopenic CKD patients. The authors used functional bars based on fruit, vegetables, extra virgin olive oil, micronized grape pomace, grape seeds and olive leaf powder derived from the recovery of waste from agri-food supply chains combined with adapted physical activity for 12 weeks. These ingredients were selected to evaluate the possible positive effects of foods rich in polyphenols in patients with CKD. This combination was helpful to counteract several CKD-related complications, such as arterial hypertension and uremic sarcopenia, and improve the CKD patients’ quality of life [115] (Table 1).

#### 5.5.3. Sulforaphane

Sulforaphane is a bioactive compound present in cruciferous vegetables and several beneficial functions have been observed in CKD. Extensive literature has shown that the main route of action is activation of the transcription nuclear factor erythroid 2 (Nrf2), which has a key role in the antioxidant response. As a result, sulforaphane protects against mitochondrial damage and helps normalize the gut microbiota [116].

#### 5.5.4. Lycopene

Lycopene is one of the most powerful antioxidants in the diet. The Framingham Osteoporosis Study has demonstrated a positive association between the intake of lycopene, a phytonutrient of the carotenoid family, and increased BMD in lumbar vertebrae, in addition to a lower risk of non-vertebral and hip fractures at 4 years [117]. A small study evaluated the effects of lycopene and calcifediol on CKD-MBD through alkaline phosphatase, PTH, as surrogate bone markers and on oxidative stress. Tomato-derived lycopene decreased cholesterol oxidation products and, in association with daily calcifediol leads to normalization of alkaline phosphatase and PTH in elderly CKD patients, suggesting preventive effects on bone disorders [118].

Mounting evidence in support of antioxidant and anti-inflammatory nutrition in CKD leads to knowledge that can provide the foundation for the prevention of musculoskeletal disorders [7,119,120]. In a recent cross-sectional study on 2569 CKD participants from NHANES, dietary inflammatory potential was positively associated with sarcopenia in patients with CKD. The dietary inflammatory potential was calculated by the dietary inflammation index score based on dietary recall interviews, and sarcopenia was assessed by low skeletal muscle mass measured by dual-energy X-ray absorptiometry. The prevalence of sarcopenia was 19.11% of patients with CKD [121].

#### 5.5.5. Brazil Nuts

Brazil nuts are an important source of selenium, which is an essential nutrient involved in bone metabolism [95]. The role in osseous remodeling is linked with a decrease in pro-inflammatory cytokine activity and direct action on osteoblasts, as the expression of selenoproteins [122]. A recent meta-analysis demonstrated that selenium intake is associated with higher BMD and a lower risk of osteoporosis and hip fracture [123].

#### 5.5.6. Glutathione

Additionally, including foods naturally high in glutathione, like avocados, spinach, okra, and asparagus, may help decrease oxidative stress. Glutathione is one of the body’s potent antioxidants. Several human and animal studies have found that eating sulfur-rich vegetables may reduce oxidative stress by increasing glutathione levels. Sulfur is required for the synthesis of glutathione, and the main sources are animal proteins and cruciferous vegetables like broccoli, Brussels sprouts, cauliflower and kale or allium vegetables like garlic and onions. Selenium is an essential mineral and a glutathione cofactor, the best sources being meats, cottage cheese and Brazil nuts. Nrf2, a transcription factor activated by oxidative stress, plays a crucial role in protecting against the harmful effects of excessive free radicals. Deletion of Nrf2 in osteoblasts results in significantly increased reactive oxygen species production due to lower glutathione levels [85].

**Figure 2:**
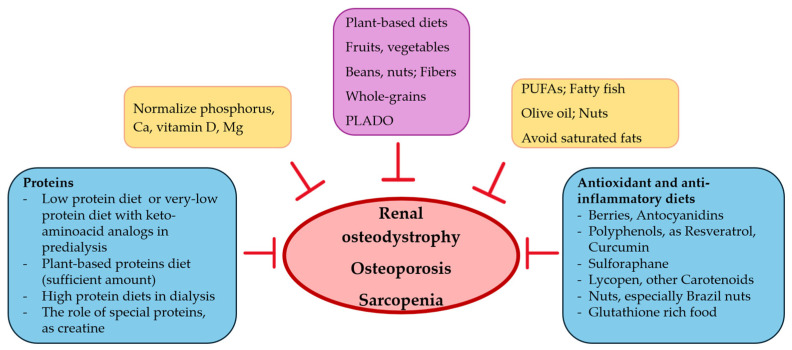
Nutritional intervention, bones and muscles in CKD.

Food has the potential to become an elixir for good and functional living in CKD, if used correctly and early in the course of the disease (Figure 2). Nephrologists should individualize dietary intervention to control osteoporosis, sarcopenia and renal osteodystrophy, according to co-morbidities and personal risk factors.

**Table 1:** Impact of nutrition on musculoskeletal complications in CKD.

Study	Design	Results
Moorthi 2015 [49]	Thirteen subjects with CKD 3–4 received an omnivorous diet containing 70% protein from plants for 4 weeks.	Over the 4-week period, urine phosphorus and titratable acid significantly decreased on the diet. No significant changes in serum FGF23, P or PTH were noted. Hand grip strength and fat-free mass did not change. A 70% plant protein diet is safe and efficacious in lowering urine phosphorus.
Lee 2020 [9]	Based on the NHANES data, BMD of different femoral areas was evaluated according to different protein diets. 12,812 subjects were assigned to: (a) <0.8 g/kg/day, (b) 0.8–1.0 g/kg/day, (c) 1.0–1.2 g/kg/day, and (d) ≥1.2 g/kg/day.	Higher-protein diets led to higher femoral BMD only in subjects without CKD. Those with low-protein diet did not reduce their femoral BMD. CKD was a risk factor for reduced BMD over the intertrochanteric bone region.
Biruete 2016 [51]	A post hoc analysis of the IHOPE trial. 138 HD patients were randomized for 12 months to placebo, protein supplementation, or protein + exercise training.	Patients ≥ 60 years old on protein supplementation maintained hip-BMD. Hip-BMD decreased in placebo group. Similar trend was observed for the femoral neck BMD. There was a lack of effect on patients < 60 years old. There was no effect of protein supplementation on body composition or blood markers of bone metabolism (Ca, P, and PTH) in either age group. In conclusion, the intradialytic protein supplementation attenuated the decrease in hip-BMD, a predictor of fractures, in older HD patients.
Umakanthan 2021 [52]	Australian single-center cohort of 39 maintenance HD patients. Muscle mass, strength and function were evaluated using bioimpedance spectroscopy, hand grip dynamometer and the timed up and go test, respectively.	The prevalence of sarcopenia was 18%. Sarcopenia was associated with low serum albumin and low phosphate levels. Low serum albumin and phosphate, as markers of protein malnutrition, resulted as significant risk factors for muscle wasting.
Marini 2024 [56]	An exploratory 1-year, balanced, double-blind study on 40 HD patients assessed the effect of creatine supplementation on body composition, and malnutrition–inflammation score was evaluated. The follow-up period was 1-year.	Creatine supplementation in HD patients for 1 year increased fat-free mass and skeletal muscle mass, associated with an increase in intracellular water, and it did not attenuate the malnutrition–inflammation score.
Gutierrez 2015 [63]	10 individuals were fed a diet providing 1000 mg of phosphorus daily using low-additive diet for 1 week, followed by a diet containing identical food items but additive-enhanced. Parallel studies were conducted in animals fed low- and high-phosphorus diets for 5 or 15 weeks. The impact of phosphorus-rich additives on bone was tested.	After an additive-enhanced diet, healthy individuals, but also mice had modified bone biomarkers, FGF23, osteopontin, and osteocalcin levels increased and sclerostin decreased. The BMD decreased in mice.
Sakaguchi 2018 [70]	The study was conducted on a nationwide database with 113,683 patients undergoing HD in Japan with no history of hip fracture. The influence of serum magnesium (Mg) on the incidence of hip fractures was evaluated on 2-year follow-up.	2% new hip fractures The incidence rate was higher among patients in the lower quartiles of serum Mg. Lower serum Mg levels were significantly associated with an increased risk of hip fracture. The risk of hip fracture was not elevated in HD patients with mild hypermagnesemia. The population-attributable fraction of serum Mg level for incident hip fractures was 13.7% which was much higher than that of serum Ca, P, and PTH levels.
Sharma 2022 [79]	The osteoprotective effect of diosmin, a citrus-derived bioflavonoid, was tested in CKD rats.	FGF23 and PTH were increased in CKD and diosmin suppressed both. CKD reduced bone mass and deteriorated the microarchitecture of trabecular bones, and diosmin maintained both at control levels. Bone formation and strength were impaired in CKD and diosmin maintained these levels at control levels.
Ryu 2021 [80]	Cross-sectional study, 68 participants with ADPKD. Muscle strength was assessed based on handgrip strength. The relationship between DASH diet and muscle strength was tested.	27.9% had low handgrip strength. Higher adherence to DASH diet was associated with low risk of reduced handgrip strength. The DASH diet can be considered as a nutritional strategy to maintain muscle strength and prevent sarcopenia in ADPKD patients
Barreto 2014 [86]	A post hoc analysis of a study on bone biopsy findings tested the relationship between indoxyl sulfate levels and bone formation rate in a group of 49 predialysis CKD patients.	The study found positive correlation between indoxyl sulfate levels and bone formation rate, osteoblast surface area, osteoid volume, and bone fibrosis volume.
Da Cruz 2024 [96]	Male Wistar rats were assigned to the following groups: sham, Nx, nephrectomized rats, and NxBN, nephrectomized rats and an enriched diet with 5% Brazil nut. Body composition parameters were obtained by DXA.	The NxBN group exhibited a significantly higher total body BMD than the Nx group. Brazil nut-enriched diet modulated BMD in CKD experimental model.
Molinari 2024 [97]	A cross-sectionally study evaluated the associations between frailty, malnutrition–inflammation syndrome and circulating inflammatory cytokines in 115 older individuals with advanced CKD.	Protein energy wasting was associated with frailty, as a manifestation of sarcopenia.
Murillo Ortiz 2019 [114]	A randomized, double-blind, placebo-controlled trial on 40 HD patients with iron overload which received combined supplementation with curcumin and resveratrol for 12 weeks	The treated group recovered bone and muscle mass.
Marrone 2024 [115]	40 CKD patients received functional foods (food bars from grape seed, grape pomace and olive leaf powders) and adapted physical activity training for 12 weeks. The progression of CKD-related comorbidities was evaluated.	This combination can help counteract uremic sarcopenia as well as arterial hypertension, dyslipidemia and improve the CKD patients’ quality of life.
Mansouri 2024 [82]	Cross-sectional study evaluated the association between pro-vegetarian dietary pattern and the risk of protein-energy wasting (assessed by low protein intake, low body and muscle mass, low albumin levels) and sarcopenia (low muscle mass, strength and function) in 109 CKD patients.	Greater adherence to pro-vegetarian diets was negatively associated with the odds of protein-energy wasting, but no association was shown between these diets and the odds of sarcopenia.
Carluccio 2016 [118]	In octogenarians, nonagenarians and centenarians with predialysis CKD, vitamin D deficiency and abnormal ALP, PTH blood values, the effects of daily lycopene supplementation on blood oxysterols as markers of oxidative stress were evaluated. The effects of calcifediol administration together with daily lycopene supplementation on PTH and ALP blood concentrations were also investigated.	Tomato-derived lycopene decreased cholesterol oxidation products. Calcifediol and lycopene were associated with normalization of ALP and PTH, suggesting preventive effects on bone disorders.
Huang 2022 [121]	The study was cross-sectional on 2569 CKD participants from NHANES. The dietary inflammatory potential was calculated by the dietary inflammation index score based on dietary recall interviews. Sarcopenia was assessed by low skeletal muscle mass measured by DXA.	The prevalence of sarcopenia was 19.11% of patients with CKD. The dietary inflammatory potential was positively associated with sarcopenia in patients with CKD.

Abbreviations: CKD, chronic kidney disease; FGF23, fibroblast growth factor 23; P, phosphorus; PTH, parathyroid hormone; NHANES, National Health and Nutrition Examination Survey; BMD, bone mineral density; HD, hemodialysis; Ca, calcium; Mg, magnesium; ADPKD, autosomal dominant polycystic kidney disease; DASH, Dietary Approach to Stop Hypertension; ALP, alkaline phosphatase; DXA, dual-energy X-ray absorptiometry.

The publication of the newest controversial conference on CKD-MBD conclusions emphasizes the importance of bone complications and proposes two clinical syndromes: CKD-associated osteoporosis, encompassing increased fracture risk in patients with CKD, and CKD-associated cardiovascular disease, including vascular calcification [124].

## 6. Conclusions

Musculoskeletal disorders are increasingly more prevalent as CKD progresses to more advanced stages. There is a global recognition of the importance of preventing and mitigating CKD-MBD, osteoporosis and sarcopenia in the CKD population given the goal of extending a life with quality and without fractures, frailty or hospitalization. There is an increasing number of health professionals stating that the management of CKD should emphasize the role of diet. Traditional renal diets became questionable, as new evidence points to the benefits of nutrients that were previously restricted. This review highlights the urgent need for intensive efforts to improve lifestyle quality as a strategy for preventing and diminishing the burden of CKD-MBD, as we aim to have less disabled CKD persons with renal osteodystrophy. In future studies, it should be confirmed whether a specific nutritional intervention may prevent deterioration in bone strength and sarcopenia in the CKD population. Vitality is a goal for people with CKD and it is achievable, as there is evidence that food may act as a geroprotector and may modulate bone and muscle functionality. Using tailored precision nutrition approaches with or without other beneficial strategies may help prevent and treat CKD-MBD and osteosarcopenia. 

## Data Availability

Not applicable.
